# Supplementary material for: Diet, Digestion, and the Dietitian: A Survey of Clinicians’ Knowledge, Attitudes and Practices to Advance the Treatment of Gastrointestinal Disturbances in Individuals with Anorexia Nervosa
Source: J Clin Med. 2022 Sep 30;11(19):5833. doi: 10.3390/jcm11195833 (PMC9573322; doi:10.3390/jcm11195833)
Supplement: Supplementary file 1 [file jcm-11-05833-s001.zip › jcm-1895184-supplementary.pdf]

Supplementary File S1: AN-GI KAPS Survey (Final version)

Section 1: Respondent Information and demographics

S1Q1. What is your gender?

S1Q2. Are you *eligible* for registration as an Accredited Practising Dietitian (APD)?

S1Q3. Do you currently treat patients with a diagnosis of anorexia nervosa?
or: Have you recently treated patients with a diagnosis of anorexia nervosa?

Section 2: Clinical experience

S2Q1. For how many years have you been working as a Dietitian?
- [Free text] years

S2Q2. For how many years have you been treating patients with anorexia nervosa?
- [Free text] years

S2Q3. In which settings do you treat patients with anorexia nervosa? (Select all that apply)
- General medical hospital
- Specialised inpatient eating disorder unit
- Specialised inpatient psychiatric facility
- Outpatient facility or program
- Private practice
- Online / Telehealth
- Research
- Other [please describe]

S2Q4. What proportion of your time working as a Dietitian is spent with patients with anorexia nervosa?
- 0-10%
- 10-25%
- 25-50%
- >50%

S2Q5. Would you classify your service as rural or urban?
- Urban
- Rural

S2Q6.Any other important details about your work setting?
- [Free text]

Section 3: Patient description

Please answer the following questions about patients with anorexia nervosa only. The term anorexia nervosa will be used to include both anorexia nervosa and atypical anorexia

S3Q1. What age group do you treat? (select all that apply)

- Children (<16 years old)
- Young adults (16-18 years old)
- Adult (18+ years old)

S3Q2. What feeding methods do you use with your patients? (Select all that apply)
Definitions:
High energy high protein liquid supplements include Ensure, Fortisip.
High energy high protein powders include Sustagen, protein powders.

- Foods
- High energy high protein liquid supplements
- High energy high protein powders/additives
- Enteral feeding (i.e. Nasogastric feeding, PEG)
- Total Parenteral Nutrition
- Other

S3Q3. Do you aim to meet nutrient requirements by food alone? (i.e. not using any additional supplements)

- None of the time
- Some of the time
- Not sure
- Most of the time
- All of the time

Section 4: Training

S4Q1. I have received formal dietetic training in the treatment of patients with anorexia nervosa and co-occurring gastrointestinal disturbances. This may include during University studies or other formal training.

- Yes
- No
- Unsure

S4Q1b. If yes, please describe the type of training: (select all that apply)

- University content (within dietetics study)
- University content (within content other than dietetics)
- Training provided by a professional organisation (e.g., ANZAED, DA)
- Other

S4Q2. I have received formal dietetic training in the treatment of patients with anorexia nervosa and co-occurring gastrointestinal disturbances. This may include during University studies or other formal training.

- Yes
- No
- Unsure

S4Q2b. If yes, please describe the type of training: (select all that apply)

- University content (within dietetics study)
- University content (within content other than dietetics)
- Training provided by a professional organisation (e.g., ANZAED, DA)
- Other

S4Q3. I have received formal training in psychological therapies:
- Yes
- No
- Unsure

S4Q3b. If yes, which formal training have you completed? (Select all that apply)
- Family-Based Therapy (FBT)
- Cognitive Behavioural Therapy for Eating Disorders (CBT-E)
- Motivational Interviewing
- Dialectical Behaviour Therapy (DBT)
- Schema Therapy
- Specialist Supportive Clinical Management (SSCM)
- Interpersonal Therapy (IPT)
- Other
- Please describe [Free text]

Section 5. Knowledge

S5Q1. Research has shown people with anorexia nervosa experience Functional Gastrointestinal Disorders (FGIDs) how often compared to the general population?
- Much more often than general population *[correct answer]*
- Somewhat more often than general population
- As often as the general population
- Less often than the general population
- A lot less often than the general population

S5Q2. Functional GI Symptoms are classified by: (select 1 option)
- Medical imaging
- Exclusion diet
- Biochemical markers
- Patient-reported symptoms or history of symptoms *[correct answer]*
- Endoscopy
- None of the above

S5Q3. Presence of GI symptoms during childhood is a risk factor for later development of an eating disorder
- True *[correct answer]*
- False
- Unsure

S5Q4. Functional GI symptoms, as for all gastrointestinal disorders, are diagnosable by a biomarker or medical imaging
- True
- False *[correct answer]*
- Unsure

S5Q5. Gastrointestinal disturbances persist after inpatient treatment
- Always
- Often *[correct answer]*

- Sometimes
- Rarely
- Never

S5Q6. Gastrointestinal disturbances may (Select all that apply):
- Interfere with body image *[correct answer]*
- Perpetuate engagement in eating disorder behaviours *[correct answer]*
- Effect quality of life *[correct answer]*
- Have led to the development of the eating disorder *[correct answer]*
- Interfere with treatment engagement *[correct answer]*

S5Q7. According to research, what percentage of individuals with an eating disorder meet diagnostic criteria for at least 1 FGID?
- > 90% *[correct answer]*
- 70%
- 50%
- < 30%

S5Q8. Are you familiar with diagnostic tools for assessing FGIDS? (E.g. ROME Criteria, Manning Criteria, Kruis Scale).
- Yes
- No

S5Q9. What do you believe is needed to improve dietitian knowledge around the intersection of GI disturbances and eating disorders?
- [Free text]

Section 6. Attitudes

S6Q1. In Functional Gastrointestinal Disorders, what does 'functional' mean to you?
- Symptoms likely to have a psychosomatic basis, probably representing somatization of psychological disturbance
- Imaginary symptoms (existing only in the patients' mind) and possibly used for secondary gain
- A real GI disorder which is currently unexplained and poorly understood

S6Q2. I believe gastrointestinal disturbances play a role in the following aspects of anorexia nervosa. (Select all that apply)
- Pathogenesis
- Engagement in eating disorder behaviours
- Engagement in treatment
- Response to treatment
- Medical complications
- Intestinal microbiota composition
- Achieving recovery
- Quality of life
- Do not play a role in any of the above
- Other, please describe [Free text]

S6Q3. I believe the dietitian should assist in management of gastrointestinal disturbances

- Strongly disagree
- Somewhat disagree
- Neither agree nor disagree
- Somewhat agree
- Strongly agree

S6Q4. Assessing, diagnosing and treating gastrointestinal disturbances is within my scope of practice as a Dietitian

- Strongly disagree,
- Disagree
- Neutral
- Agree
- Strongly agree

S6Q5. Within the medical team, I believe treatment of gastrointestinal disturbances is the responsibility of: (Please select all that apply)

- Psychiatrist
- Nurse
- Physician
- Dietitian
- Physiotherapist
- Gastroenterologist
- Other: [Free text]

S6Q6. I believe patients perceive gastrointestinal disturbances as a barrier to achieving recovery

- Strongly disagree
- Somewhat disagree
- Neither agree nor disagree
- Somewhat agree
- Strongly agree

S6Q7. I believe gastrointestinal disturbances are a symptom of anorexia nervosa and will resolve over time, without specific treatment

- Strongly disagree
- Somewhat disagree
- Neither agree nor disagree
- Somewhat agree
- Strongly agree

S6Q8. I believe gastrointestinal disturbances: (Select all that apply)

- Are psychosomatic
- Are a symptom of disordered eating
- Are a symptom of disordered attitudes toward food and eating
- Can encourage eating disorder behaviours
- Can complicate treatment
- Are a conditioned response to feared foods
- Other > Please describe

S6Q9. I believe gastrointestinal disturbances have major consequences for a patient's quality of life

- Strongly disagree
- Somewhat disagree
- Neither agree nor disagree
- Somewhat agree
- Strongly agree

S6Q10. If patients report gastrointestinal disturbances, I modify treatment

- None of the time
- Some of the time
- Not sure
- Most of the time
- All of the time

If 'None of the time' please describe why you don't modify treatment: [free text]

S6Q11. I believe my view and my patients' view of their experience with gastrointestinal disturbances is generally similar?

- Strongly disagree
- Somewhat disagree
- Neither agree nor disagree
- Somewhat agree
- Strongly agree

Section 7. Practices

S7Q1. I am confident in my ability to treat co-occurring gastrointestinal disturbances and anorexia nervosa

- Strongly disagree
- Somewhat disagree
- Neither agree nor disagree
- Somewhat agree
- Strongly agree

S7Q2. I expect gastrointestinal disturbances to improve with weight restoration

- Strongly disagree
- Somewhat disagree
- Neither agree nor disagree
- Somewhat agree
- Strongly agree

S7Q3. Education about the relationship between gastrointestinal disturbances and eating disorder should be provided by the dietitian

- Strongly disagree
- Somewhat disagree
- Neither agree nor disagree
- Somewhat agree
- Strongly agree

S7Q4. I routinely screen patients for functional GI disorders or disturbances

- None of the time
- Some of the time
- Not sure
- Most of the time
- All of the time

S7Q4b. What do you use to screen patients for functional GI disorders?
- ROME Criteria
- Manning Criteria
- Kruis Criteria
- Other [Free text]

S7Q5. Strategies I use to address GI disturbances include: (Select all that apply)
- Refer to a gastroenterologist
- Medication advice
- Suggest use of peppermint oil
- Low FODMAP diet
- Exclusion of particular food groups (e.g. Dairy, Gluten, etc.)
- Suggest use of fibre supplement
- Suggest use of probiotics
- Over the counter nutrition supplements > provide details: [Free text]
- Suggest use of anti-diarrhoeal agents
- Suggest advice on meal timing
- Provide advice on pre- or post-meal activities
- Gut-focused hypnotherapy
- Refer to psychologist or counsellor
- Suggest breathing techniques
- Mindful eating techniques
- Provide information about GI disturbances and ED
- Other > Please describe [Free text]
- I don't use any specific strategies

S7Q6. I provide education to patients about gastrointestinal function and gut health:
- None of the time
- Some of the time
- Not sure
- Most of the time
- All of the time

S7Q6b. What type of gastrointestinal education do you provide to your patients? (Select all that apply):
- Psychoeducation
- Reassurance
- Suggest consultation with paediatrician
- Suggest consultation with psychologist
- Suggest consultation with GP
- Suggest consultation with gastroenterologist
- Other > Please describe [Free text]

S7Q7. In general, I feel confident approaching a patient with co-occurring anorexia nervosa and gastrointestinal disturbances

- Strongly disagree
- Somewhat disagree
- Neither agree nor disagree
- Somewhat agree
- Strongly agree

Section 8. Knowledge, attitudes and practices: Gut Microbiome

S8Q1. In regard to the gut microbiome… (Select all that apply)

- I am knowledgeable
- I am interested
- I am sceptical
- I want to learn more
- None of the above
- Other > [free text]

S8Q2. I believe the gut microbiome may play a role in: (Select all that apply)

- Behaviour
- Body weight
- Food choices
- Metabolism
- Treatment success
- Recovery
- Gastrointestinal disturbances
- Psychological state
- None of the above
- Other > [free text]

S8Q3. I encourage my patients to test their gut microbiome with stool analysis

- Strongly disagree
- Somewhat disagree
- Neither agree nor disagree
- Somewhat agree
- Strongly agree

Section 9: Other comments

S9Q1. Please share any other comments about anorexia nervosa and gastrointestinal disturbances:

- [Free text]
